# Precision Neuromodulation Treatment Reverses Motor and Cognitive Slowing After Stroke: Clinical and Neurophysiological Evidence

**DOI:** 10.3390/jcm15020713

**Published:** 2026-01-15

**Authors:** Gianna Carla Riccitelli, Riccardo Gironi, Edoardo Ricci, Pamela Agazzi, Daniela Distefano, Chiara Zecca, Claudio Gobbi, Alain Kaelin-Lang

**Affiliations:** 1 Neurocenter of Southern Switzerland, EOC, 6900 Lugano, Switzerland; riccardo.gironi@eoc.ch (R.G.);; 2 Faculty of Biomedical Sciences, Università della Svizzera Italiana, 6900 Lugano, Switzerland; 3 Department of Neurology, Inselspital, Bern University Hospital, 3010 Bern, Switzerland

**Keywords:** stroke rehabilitation, repetitive transcranial magnetic stimulation, cognitive training, quantitative EEG, neurorehabilitation

## Abstract

**Background/Objectives:** Chronic psychomotor and cognitive slowing after stroke can persist despite standard rehabilitation, especially in young adults with subcortical injuries. Innovative, integrated interventions are crucial for patients who have reached a plateau in their rehabilitation. We present a case of a 41-year-old male with chronic psychomotor and cognitive slowing following a left lenticulostriate infarction (NIHSS score = 5 at onset), who had plateaued after conventional rehabilitation. **Methods:** Over 4 weeks the patient underwent 20 sessions of a multimodal approach including high-frequency repetitive transcranial magnetic resonance stimulation over the supplementary motor area and bilateral temporo-parietal junctions and simultaneous computerized cognitive training targeting attention and executive function. Both motor and cognitive assessments, along with quantitative EEG (qEEG) evaluations, were conducted before and after the treatment. **Results:** At the end of treatment, the patient showed significant clinical improvement: speed and coordination in upper extremities (Finger Tapping Test) increased by 66% (dominant hand) and 74% (non-dominant hand), while finger dexterity (Nine-Hole Peg Test) increased by 25% (dominant hand) and 19% (non-dominant hand). Cognitive scores improved in alertness (58%), visual exploration (25%), and flexibility (24%), while divided attention remained stable. qEEG investigation showed increases in alpha (79%), gamma (33%), and beta (10%) power, with topographic shifts in the stimulated regions. **Conclusions:** These findings highlight the feasibility of combining targeted rTMS and cognitive training to enhance neuroplasticity in the chronic phase of stroke. Clinical recovery was accompanied by normalized cortical rhythms, suggesting qEEG biomarkers may be useful for tracking treatment response. Multimodal precision neurorehabilitation may offer a path forward for patients with persistent cognitive–motor deficits post-stroke.

## 1. Introduction

Cervical arterial dissections (CADs) account for approximately 25% of ischemic strokes in individuals under the age of 45, positioning CAD as one of the most common causes of stroke in young adults [1].

This population, typically defined as individuals under 50 years of age, represents 10–15% of all stroke cases, with incidence rates of approximately 18 per 100,000 person-years in Europe [2].

The trajectory of recovery in these patients follows distinct temporal phases: acute (first 24 h up to 7 days), subacute (up to 6 months), and chronic (beyond 6 months post-onset) [3]. While spontaneous neurological improvement is most robust during the acute and early subacute periods, therapeutic options for chronic-phase cognitive deficits remain scarce despite persistent impairment in many survivors [4]. This gap is particularly critical for younger patients facing decades of potential disability.

Beyond the acute vascular event, many patients develop lasting neurological sequelae affecting both motor and cognitive domains [5]. Motor deficits such as bradykinesia frequently co-occur with post-stroke cognitive impairment (PSCI), defined as any cognitive decline following stroke, ranging from single-domain deficits to dementia, affecting up to 50% of survivors within the first year [6]. This dual burden significantly compromises functional independence and return-to-work capacity, even in the absence of overt physical disability [7].

Current rehabilitation approaches often struggle to address the multidimensional nature of these deficits. Nearly 50% of stroke patients continue to experience moderate to severe limitations despite conventional neurorehabilitation protocols [8].

Growing evidence supports the use of non-invasive brain stimulation, particularly repetitive transcranial magnetic stimulation (rTMS), to enhance neural recovery by modulating cortical excitability and rebalancing disrupted network dynamics. Stimulation of the primary motor cortex and supplementary motor area (SMA) has been shown to improve motor planning, coordination, and bradykinesia through facilitation of cortico-cortical and cortico-subcortical pathways [9,10]. Additionally, theta-burst stimulation targeting posterior parietal and temporo-parietal regions can modulate the ventral attention network, normalizing aberrant connectivity patterns and improving visuospatial attention and processing speed [11].

rTMS combined with cognitive training has shown promise in improving cognitive function and activities of daily living in patients with post-stroke cognitive impairment, as evidenced by a recent systematic review and meta-analysis [4], which reported positive effects on global cognition, executive function, and working memory compared with control conditions. However, most existing studies focus on earlier phases of stroke recovery and on older populations [4].

Despite this promise, few studies have systematically explored these integrative protocols in younger stroke patients during chronic phase or evaluated outcomes using neurophysiological biomarkers.

Addressing these gaps, this pilot study uniquely applies a multimodal neuromodulation intervention combining simultaneous targeted rTMS with computerized cognitive training (CCT) in post-stroke patients with motor and cognitive persistent symptoms.

Standardized cognitive and functional metrics and quantitative electroencephalography (qEEG) were employed to identify pre- and post-intervention brain dynamics and behavioral changes, respectively. This clinical case explores a novel approach to targeting disrupted motor–cognitive networks through synergistic neuromodulation and adaptive training.

## 2. Materials and Methods

### 2.1. Case Description

A 41-year-old male, previously in good general health, presented to the emergency department after experiencing a sudden-onset, unusual headache in the left temporo-parietal region the previous evening. He had taken Dafalgan 500 mg. Later that night, his wife heard a thud and entered the bedroom where she found him on the floor, poorly responsive, with speech difficulties. When attempting to help him back into bed, she noticed weakness in the right lower limb. Emergency services were called. Upon their arrival, the patient had a Glasgow Coma Scale of 12 and presented with slower responsiveness, right facial droop and dysarthria. On arrival at the emergency department his condition showed improvement. He remained slowed, partial disoriented in time and space with a right-sided central facial palsy and dysarthria, yielding a National Institutes of Health Stroke Scale (NIHSS) score of 5. The patient had a prior diagnosis of glaucoma, managed with timolol ophthalmic drops.

A brain computed tomography (CT) without contrast showed no hemorrhage or early signs of ischemia, while CT-angiography demonstrated a focal stenosis of the distal extra-cranial segment of the left internal carotid artery and a sub-occlusive thrombus at the M1 bifurcation with a large area of ischemic penumbra in the left middle cerebral artery (MCA) territory.

Given the patient’s stable clinical presentation and spontaneous neurological improvement, intravenous thrombolysis was not administered because symptom onset exceeded the therapeutic window (<4.5 h). Mechanical thrombectomy and carotid stenting were also not indicated given the further spontaneous improvement. Medical management with dual antiplatelet therapy (DAPT) was initiated.

Subsequent brain magnetic resonance (MR) showed an acute ischemic lesion in the left striatum and further smaller foci of ischemia in the precentral gyrus, the insula, the frontal operculum and the anterior limb of the internal capsule. Further MR details are reported in Figure S1.

During a one-week hospitalization in the Stroke Unit, the patient remained alert and oriented. Ideomotor slowing was pronounced. Voluntary movements of the right upper and lower limbs were markedly slowed, with inefficient and uncoordinated motor execution. The right facial palsy persisted, while motor strength was preserved in all four limbs. No aphasia, dysarthria, ophthalmoparesis, limb ataxia, or sensory deficits were observed.

A comprehensive pharmacological regimen was initiated to manage ischemic insult and reduce the risk of further complications.

Following clinical stabilization, the patient was transferred to a rehabilitation center, where he participated in a structured, multidisciplinary program from January to April 2024. Interventions included physiotherapy, targeted motor rehabilitation with the Therabike, hydrokinesiotherapy, and visual-motor re-education.

Neuropsychological rehabilitation involved cognitive training via a combination of computerized and paper-based exercises (Rehacom, Hasomed GmbH; COCARE system, Dividat).

Group speech therapy targeted dysarthrophonia, hypophonia, and slowed speech. Occupational therapy focused on enhancing independence in activities of daily living (ADLs), including tasks like silk painting and meal preparation, to improve coordination, dexterity, and upper-limb strength. The patient continued multidisciplinary rehabilitation at home.

During and after inpatient rehabilitation, the patient received psychological and psychiatric care, with gradual improvement of mood symptoms. A consistent improvement in the affective state was observed over time during systematic monitoring, indicating progressive mood stabilization.

At the clinical visit of follow-up (July 2024), the patient was alert, oriented, and cooperative. However, notable psychomotor slowing persisted. Speech assessment revealed mild hypophonia and reduced verbal fluency, without anomia or dysarthria. Gait was stable, with preserved pendular limb movements and the ability to walk on heels, toes, and in tandem. Mild bradykinesia was observed in repetitive hand movements, including finger tapping, pronation–supination, and toe tapping. The Pull Test was positive, indicating retropulsion and postural instability.

The patient’s treatment regimen included clopidogrel, valsartan/hydrochlorothiazide, timolol, and domperidone. Venlafaxine (75 mg/day) was initiated shortly after stroke onset to manage post-stroke adjustment symptoms. The dose remained unchanged throughout the intervention period and follow-up.

Levodopa/benserazide, initiated one month before the follow-up (June 2024), was discontinued due to lack of clinical efficacy.

**Figure 1:**
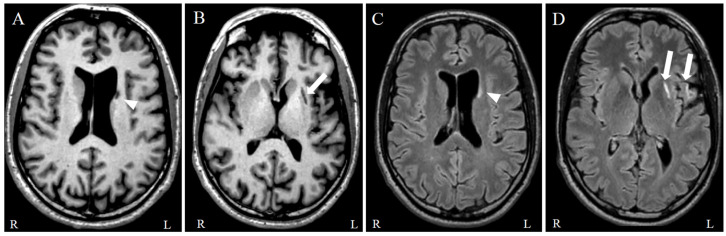
Brain MRI images acquired seven months after the ischemic event. (**A**,**B**) T1-weighted axial images demonstrate an ex-vacuo dilatation of the left lateral ventricle with atrophy of the left caudate nucleus (arrowhead) and a focal area of encephalomalacia with atrophy of the left putamen (arrow). (**C**,**D**) Axial FLAIR images show areas of high signal in the left caudate nucleus (arrowhead), putamen and operculum (double arrows). MRI, magnetic resonance imaging; FLAIR, Fluid Attenuated Inversion Recovery.

Follow-up MR showed gliotic changes in the left striatum and frontal operculum consistent with prior ischemic injuries (Figure 1).

Despite meaningful improvements, persistent psychomotor and ideomotor slowing continued to limit functional recovery. In light of these deficits, the neurology team proposed an off-label therapeutic strategy involving the simultaneous delivery of non-invasive brain stimulation and targeted cognitive training, as an adjunct to the ongoing rehabilitation protocol.

Before the intervention began, an eligibility assessment was conducted using the guidelines of the Safety Questionnaire for TMS [12], and the potential side effects were thoroughly discussed with the patient. Throughout the treatment process, no adverse effects were observed, and the patient demonstrated a positive response to the treatment.

### 2.2. Outcome Measures

Before and after neuromodulation treatment (NT), the patient undergoes a comprehensive motor–cognitive and EEG recording to investigate motor, cognitive and cortical activity changes resulting from the intervention.

#### 2.2.1. Dexterity and Coordination

Nine-Hole Peg Test (NHPT) is used to assess both motor speed and eye–hand coordination, specifically in the context of bradykinesia [13]. During the test the subject was required to place pegs, shaped like keys, into a board with randomly oriented recesses. The test was administered twice, once using the right hand and once using the left hand. The outcome measure is the time taken to complete the task in seconds for each hand. Raw scores were standardized using Z-scores based on normative data [14,15].

Finger Tapping test (FT) is used to measure upper limb dexterity and motor speed [16].

During the test the board was placed in front of the subject’s dominant (D) hand for the initial trial. The participant was instructed to tap the lever as quickly as possible with their index finger until the examiner stopped the timer. The test was conducted with alternating trials for the dominant and non-dominant (ND) hands, with each trial lasting 10 s, resulting in four trials (D-ND-D-ND). The total number of taps for each hand was recorded, and the mean score was calculated across these values [15].

For the NHPT, lower scores indicate better performance, whereas for FT, higher scores indicate better performance. Differences in NHPT and FT performances between pre- and post-NT were assessed using a percentage-based formula to quantify the relative improvement more effectively [17].

#### 2.2.2. Attentional, Speed and Flexibility

To investigate attention-executive abilities, Test of Attentional Performance (TAP) was used. It is a computerized battery designed to evaluate various components of attention and specific aspects of visual perception [17]. This tool provides a detailed analysis of different attentional processes, offering a comprehensive assessment of attentional-executive functions. For the patient, we assessed the following subscales: alertness, divided attention, visual exploration, and flexibility.

The battery employs continuous stimulus detection paradigms, enabling the evaluation of both sustained attention and inhibitory control in the presence of distracting stimuli. For this assessment tool, lower scores indicate better performance. Test results are interpreted based on normative values adjusted for the subject’s age, gender, and educational background [18].

Delta percentage-based formula was applied to describe the direction and magnitude of cognitive change [17].

### 2.3. Neurophysiological Assessment

The patient underwent a 64-channel EEG while sitting comfortably and with closed eyes in an armchair, with a duration of 15 min. EEG signals were acquired using an EEG cap (Waveguard original EEG cap, ANT Neuro, Enschede, Netherlands) with Ag/AgCl electrodes, positioned on the scalp according to the extended international 10–20 system [19]. The reference was located in Cpz, while Afz was used as the ground electrode. Impedance was maintained below 10 kΩ for every channel. A 500 Hz sampling rate was used to process the signal, with a 24-bit resolution and amplified with eego™ sports amplifier (ANT Neuro, Enschede, Netherlands).

The same procedure was performed at the end of NT.

For the qEEG analysis, signals were preprocessed using EEGLAB (v2024.2) [20] in MATLAB (R2024b; The MathWorks Inc., Natick, MA, USA). The continuous EEG recordings were segmented into 3 s epochs and underwent preprocessing, including band-pass filtering (1–40 Hz) and notch filtering (50 Hz) to remove power line interference. For artifact removal, Independent Component Analysis (ICA) was applied, followed by component classification with ICLabel [21]. Components associated with eye movements, muscle activity, cardiac signals, noise, or other non-neural sources were automatically removed. A visual inspection of the components was also performed concurrently with the automated classification. Only components with a brain signal probability exceeding 30% were retained for further analysis.

Spectral power was estimated using Welch’s method across delta (0.5–4 Hz), theta (4–8 Hz), alpha (8–13 Hz), beta (13–30 Hz), and gamma (>30 Hz) frequency bands. This standardized analysis protocol was applied identically to both pre- and post-TMS EEG recordings.

Statistical comparison of spectral metrics across treatment conditions was performed using the Wilcoxon signed-rank test. A delta percentage-based formula was applied to describe the direction and magnitude of EEG bands’ power change [22].

### 2.4. Intervention

The NT was conducted over four weeks, with the patient undergoing daily sessions (5 days per week, for a total of 20 sessions). We stimulated three cortical targets sequentially in each session: the SMA and the right and left temporoparietal junctions (TPJ).

In addition to the SMA, we applied high-frequency rTMS to the right and left TPJ in each session to engage bilateral attentional and sensorimotor networks and facilitate interhemispheric modulation [23].

#### 2.4.1. Non-Invasive Brain Stimulation: rTMS

rTMS was carried out using a Magstim Rapid2 magnetic biphasic stimulator connected with a figure-of-eight coil with a 70 mm diameter (Magstim Company, Whitland, UK) that generates 2.2 T as maximum output. Each daily session of treatment included the stimulation of 3 brain regions, SMA and bilateral TPJ.

Cortical targets were localized using MRI-based neuronavigation. The patient’s T1-weighted structural MRI was used as an anatomical reference, and target coordinates in Talairach space were imported into the SofTaxic Navigator system (EMS, Bologna, Italy). Scalp positions were estimated automatically, allowing consistent coil placement over the SMA and bilateral TPJ before each stimulation session. For each brain region, the following parameters were applied: 30 trains of 3 s delivered at 20 Hz spaced out by 20 s of no stimulation (total number of stimuli: 1800).

The entire session lasted approximately 40 min. Intensity of stimulation was set at 100% of the resting motor threshold (RMT), defined as the lowest intensity producing MEPs of >50 μV in at least five out of 10 trials in the relaxed first dorsal interosseous (FDI) muscle of the right hand [24]. RMT was assessed over the optimal stimulus site to elicit MEPs in the right FDI, termed “motor hotspot”, identified by positioning the coil approximately over the central sulcus and moving it on the scalp by 0.5 cm steps on left M1.

#### 2.4.2. Cognitive Rehabilitation: CCT

To promote active engagement of the targeted cognitive networks, we employed an online platform (BrainBooster®; brain-booster.ch), which offers clinician-customizable cognitive tasks. Its flexibility in adjusting exercise parameters to individual needs makes it particularly suitable for integration with neuromodulation protocols.

CCT was delivered concurrently with rTMS, focusing on attention, executive function, and processing speed abilities. Tasks for each NT session were selected and adapted based on the patient’s performance during the previous session. The supervising neuropsychologist tailored the cognitive training to the patient’s performance from the previous day and, if needed, made real-time adjustments during each session. An option that ensures an optimal challenge-to-skill ratio and high engagement was used throughout the protocol. Additionally, each session was preceded by approximately 15 min of trial-based practice to ensure comprehension and familiarity with the newly introduced tasks.

The combined approach (rTMS and CCT) relied on the temporary increase in cortical excitability induced by rTMS to enhance task-related network activation.

## 3. Results

### 3.1. Movement and Cognitive Findings Pre-NT

Before the neuromodulation treatment (NT) intervention, the patient showed poor performance in both the Finger Tapping (FT) test and Nine-Hole Peg Test (NHPT). The FT mean scores were 15.7 for the right hand and 15.3 for the left hand, while the NHPT mean scores were 158.5 s for the right hand and 126 s for the left hand.

A selected investigation of attention and executive abilities showed abnormal performances at Test of Attentional Performance (TAP) subtests: alertness (mean = 762 ms), visual exploration (mean = 8468 ms), and flexibility (mean = 671 ms) and divided attention (mean = 844 ms).

### 3.2. Clinical, Motor and Cognitive Changes Post-NT

At neurological visit conducted after the NT, the patient exhibited a notable clinical improvement in psychomotor slowing: repetitive hand movements were performed without bradykinesia, with mildly slowed pronation–supination, and toe tapping. Gait improved, with the ability to take longer steps while maintaining preserved pendular movements. The Pull Test was negative, suggesting improved postural stability. Although the speech had a slightly hypophonic tone, with reduced verbal fluency, a greater ability to recruit verbal responses was recorded. No anomia or dysarthria was present.

Motor function performances were significantly improved. FT scores for the right hand increased by 66% and for the left hand by 74%. A significant reduction in reaction times was found in NHPT performances, with a 25% decrease for the right hand and a 19% for the left hand, indicating faster and more coordinated actions.

Cognitively, an improvement in attentive capabilities was recorded, with a 58% increase in alertness. Performances, including executive components such as visual exploration and flexibility, improved by 25% and 24%, respectively. However, no improvement was found in divided attention performance, and execution time increased by approximately 8%. Table 1 summarizes motor and cognitive scores before and after NT.

The patient maintained stable affect and high task engagement throughout the cognitive training sessions, supporting the exclusion of active depressive symptoms as a confounding factor.

### 3.3. qEEG Findings Pre and Post NT

Before NT, spectral power showed a predominant frontal distribution in the lower frequency bands, particularly in the delta (mean = 1.423) and theta (mean = 0.705) ranges. Alpha activity (mean = 1.316) appeared more diffuse but less pronounced over posterior regions, while beta (mean = 0.317) and gamma (mean = 0.003) bands exhibited lower overall power, with a tendency toward anterior localization.

After NT, EEG measures showed significant changes in several frequency bands: alpha wave power experienced the most relevant change with a 78.9% increase (from 1.316 to 2.355, *p* < 0.001), followed by gamma wave power, which increased by 33.3% (from 0.003 to 0.004, *p* < 0.05). Beta activity showed a moderate increase of 10.4% (from 0.317 to 0.350, *p* < 0.001), while theta activity decreased by 8.5% (from 0.705 to 0.645, *p* < 0.001).

No significant change was observed in delta wave power, observing a slight decrease of 0.9% (from 1.423 to 1.410, *p* = 0.696).

**Figure 2:**
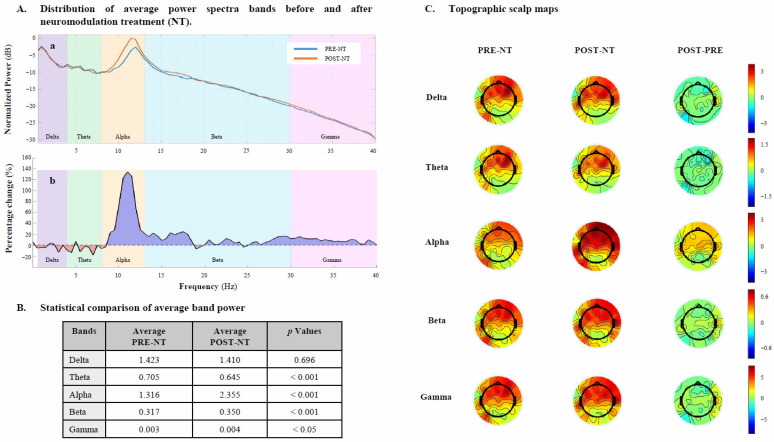
(**A**) Neurophysiological changes observed through qEEG before and after neurostimulation (NT) intervention. (**a**) Average power spectra across all frequency bands comparing the PRE- and POST-NT intervention conditions. (**b**) Difference in average power across frequency bands (POST–PRE). (**B**) Mean power values of EEG frequency bands measured PRE- and POST-NT intervention. Data normality was assessed using Shapiro–Wilk test. (**C**) Topographic scalp maps depicting the spatial distribution of EEG power across the five frequency bands at PRE-, POST-NT, and POST–PRE NT intervention.

Frequency-band changes and relative topographic distribution are shown in Figure 2.

## 4. Discussion

This case highlights the therapeutic potential of a novel multimodal, precision-guided neurostimulation strategy in a young post-stroke patient with chronic ischemic lesions in the left lenticulo-striato-capsular and fronto-operculo-insular regions (Figure 1), areas intimately involved in motor planning, initiation, and executive functioning. The dorsal striatum (putamen and caudate), which was directly affected, receives convergent glutamatergic input from the SMA and dopaminergic projections from the substantia nigra pars compacta.

Disruption of this circuitry can impair both motor output (bradykinesia, reduced dexterity) and cognitive dynamics (slowed processing, attentional deficits), due to the role of dopamine in modulating synaptic plasticity within the basal ganglia-thalamocortical loop [25,26].

These impairments were reflected in the patient’s qEEG profile, which showed elevated delta and theta activity alongside suppressed alpha and gamma power, an electrophysiological signature of reduced cortical efficiency and network dysregulation in post-stroke states (Figure 2) [27,28].

The application of excitatory rTMS over the SMA and bilateral TPJ likely acted through several complementary biological pathways to restore function. SMA stimulation is known to modulate cortico-striatal excitability and can induce dopamine release in the ipsilateral striatum [29], thereby promoting recovery of motor functions impaired by basal ganglia lesions. In preclinical models, rTMS over the SMA increases N-methyl-D-aspartate receptor expression and synaptic efficacy in motor circuits, leading to improved movement speed and planning [30,31].

In parallel, bilateral stimulation of the TPJ, a multisensory hub implicated in spatial attention, postural control, and visuomotor integration, likely contributed to improved balance, coordination, and visual exploration. The TPJ’s role in attentional reorienting and sensory-motor integration aligns with gains observed in the TAP subscales of flexibility, alertness, and visual exploration (Table 1) [32,33,34]. Previous work shows TPJ-targeted rTMS can enhance spatial awareness and improve cognitive–motor integration [35,36]. In addition, the added impact of bilateral stimulation, as high-frequency rTMS to both TPJs, has been shown to modulate interhemispheric dynamics and enhance motor training effects in post-stroke recovery [23].

Thus, by targeting both SMA and TPJ, rTMS may have facilitated network-level plasticity, engaging both motor and cognitive networks through reactivation of residual corticocortical and corticostriatal loops [37,38,39].

While rTMS modulated cortical excitability and primed neural circuits for change, concurrent CCT provided task-driven synaptic activation necessary to consolidate functional gains through Hebbian plasticity [40].

The synergistic benefits of this combined protocol are evident in both behavioral and neurophysiological domains. For instance, the relevant 58% improvement in cognitive alertness and 24% improvement in flexibility are difficult to attribute to rTMS or training alone. Prior studies suggest that neurostimulation enhances the brain’s receptivity to behavioral input, making it more likely that task-induced activation leads to durable synaptic remodeling [41,42,43].

This dual-modality protocol likely overcame the ceiling effects observed during conventional rehabilitation, enabling renewed neural plasticity in the chronic phase of stroke recovery, typically considered a plateau period [44].

The patient’s qEEG provides strong neurophysiological evidence of functional reorganization. Post-treatment, there was a marked increase in alpha (78.9%) and gamma (33.3%) power, alongside a reduction in pathological theta (8.5%), suggesting a restoration of cortical oscillatory balance (Figure 2). These spectral changes suggest an improved neural synchrony, excitability, and information processing capacity, consistent with the observed clinical recovery. Moreover, the diffuse increases in alpha and gamma power observed in this case likely reflect reorganization of distributed motor–cognitive networks, rather than being limited to local effects at the lesion site. This interpretation is supported by previous multimodal studies demonstrating that rTMS combined with cognitive training can promote widespread connectivity changes, which are closely associated with functional improvements [45,46]. These findings suggest that recovery dynamics in chronic stroke may involve network-level modulation affecting both ipsilesional and contralesional regions. The alignment of these EEG changes with improved performance on TAP and motor tests suggests that spectral metrics may serve as reliable biomarkers of recovery and target engagement [47].

The successful outcome in this case supports the rationale for precision-guided, network-targeted neurorehabilitation. By integrating structural imaging, neurophysiological biomarkers, and individualized stimulation and training protocols, clinicians can design synergistic interventions tailored to the patient’s lesion profile and residual network capacity [48,49].

Importantly, this case demonstrates that such an approach not only yields multidomain clinical benefits across motor dexterity, executive function, and attentional speed but also facilitates meaningful occupational reintegration in a working-age individual. Given the profound personal and societal costs of stroke in young adults, interventions that extend beyond impairment-level improvements to restore function and productivity are of critical importance [8].

Despite the promising results, the single-case nature of this study, combined with the absence of a control condition, constrains our ability to determine the independent contributions of rTMS and CCT. Future randomized controlled trials should explore the dose–response characteristics of each modality, investigate long-term sustainability of gains, and determine whether neurophysiological markers such as EEG can serve as predictors of treatment response.

## 5. Conclusions

This case demonstrates that combining simultaneous rTMS and CCT can lead to significant improvements in motor and cognitive function in chronic stroke. The intervention facilitated network-level reorganization, as evidenced by neurophysiological changes and clinical outcomes.

These findings provide preliminary support for the potential of combining simultaneous rTMS and CCT to enhance motor and cognitive function in chronic stroke; however, larger studies are needed to confirm efficacy and define optimal parameters.

## Figures and Tables

**Table 1:** Motor and cognitive score performances before and after NT.

Domain	Test	Score Type	Pre-NT Mean (SD), [Min–Max]	Post-NT Mean (SD), [Min–Max]	Delta (Absolute Change) ^§^	Percentage Change ^#^
**Motor**	Finger Tapping (Right, Dominant hand)	*Number taps*	15.7 (2.08) [14–18]	26 (2.00) [24–28]	10	+66%
	Finger Tapping (Left, Non-dominant hand)	*Number taps*	15.3 (1.53) [14–17]	26.7 (0.58) [26–27]	11	+74%
	Nine-Hole Peg Test (Right, Dominant hand)	*Time (s)*	158.5 (7.78) [153–164]	118.5 (7.78) [113–124]	−40	−25%
	Nine-Hole Peg Test (Left, Non-Dominant hand)	*Time (s)*	126 (14.14) [116–136]	102.5 (4.95) [99–106]	−24	−19%
**Cognitive**	TAP-alertness	*Reaction time (ms)*	762 (154)	320 (58)	−442	−58%
	TAP-visual exploration	*Reaction time (ms)*	8468 (2374)	6339 (1910)	−2129	−25%
	TAP-flexibility	*Reaction time (ms)*	671 (129)	511 (87)	−160	−24%
	TAP-divided attention	*Reaction time (ms)*	844 (188)	913 (215)	69	+8%

**Legend and notes: **^§^, (Post-NT) − (Pre-NT); ^#^, ((Post-NT − Pre-NT) ÷ Pre-NT) × 100; NT, Neuromodulation Treatment; SD, Standard Deviation; ms, milliseconds; s, seconds.

## Data Availability

The data presented in this study are available upon reasonable request from the corresponding author. The data are not publicly available owing to privacy and ethical restrictions.

## Supplementary Materials

The following supporting information can be downloaded at: https://www.mdpi.com/article/10.3390/jcm15020713/s1, Figure S1. CT and MRI exams performed during emergency and stroke department recovery.
