# Supplementary material for: Precision Neuromodulation Treatment Reverses Motor and Cognitive Slowing After Stroke: Clinical and Neurophysiological Evidence
Source: J Clin Med. 2026 Jan 15;15(2):713. doi: 10.3390/jcm15020713 (PMC12842392; doi:10.3390/jcm15020713)
Supplement: Supplementary file 1 [file jcm-15-00713-s001.zip › jcm-4048853-supplementary.pdf]

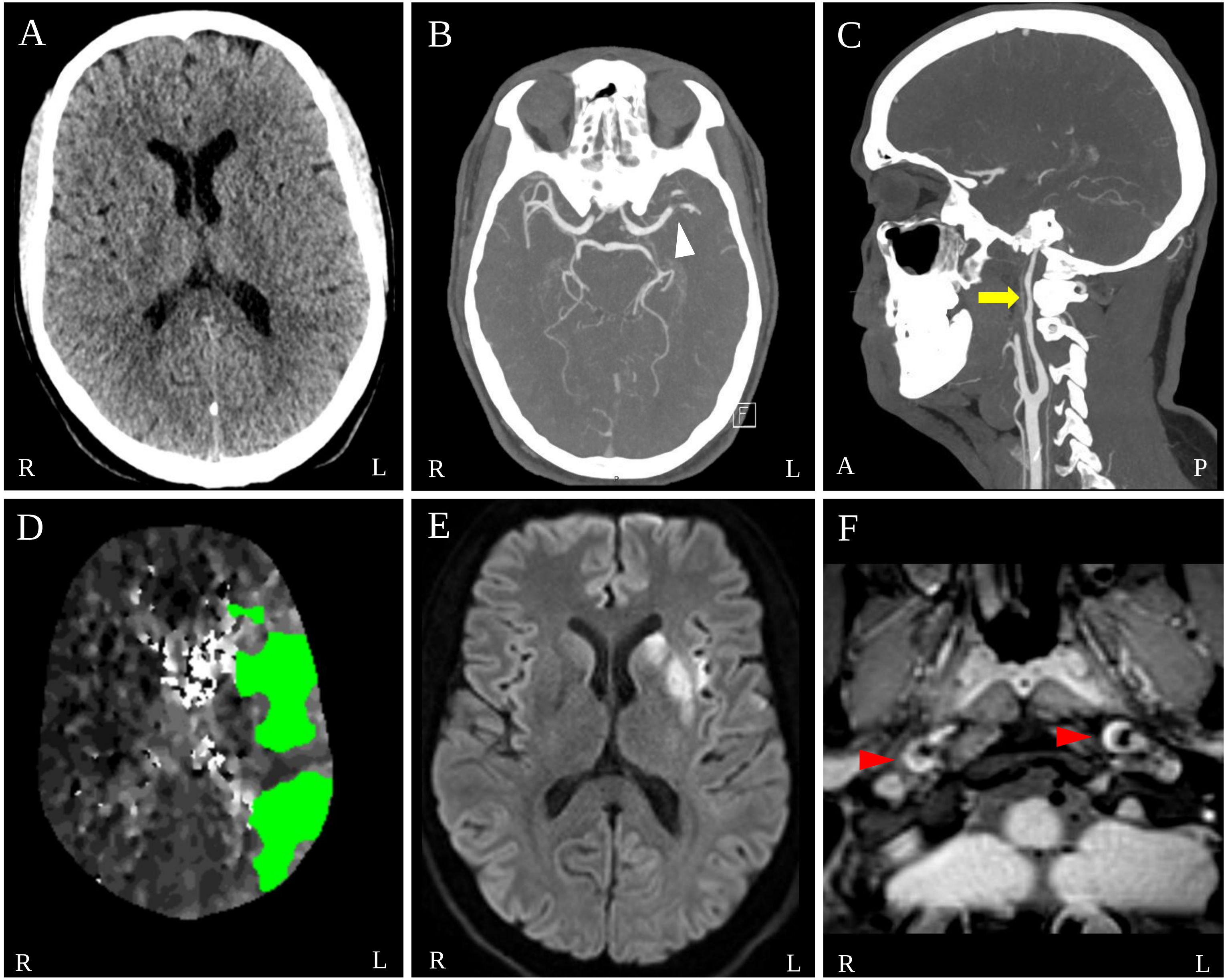

Figure S1. CT and MRI exams performed during emergency and stroke department recovery.

A. Axial CT head image without contrast shows no hemorrhage or early signs of ischemia.

B-C. Axial and sagittal MIP CT-Angiography images demonstrate a sub-occlusive thrombus located at the left M1 bifurcation (white arrow) and vessel wall irregularities of the left internal carotid artery (yellow arrow)

D. Tmax map obtained with software RAPID shows a large area of ischemic penumbra in the left MCA territory.

E. Axial diffusion-weighted image revealed areas of restricted diffusion in the striatum and the insula on the left side. **F.** Axial T1-weighted-fat-sat image shows bilateral high signal crescent sign in the internal carotid arteries indicating a bilateral dissection. CT, computed tomography; MIP, maximum intensity projection; RAPID, real-time automated processing of infarct diagnosis; MCA, middle cerebral artery.
